# A Proteinaceous Alpha-Amylase Inhibitor from Moringa Oleifera Leaf Extract: Purification, Characterization, and Insecticide Effects against *C. maculates* Insect Larvae

**DOI:** 10.3390/molecules27134222

**Published:** 2022-06-30

**Authors:** Aida Karray, Mona Alonazi, Raida Jallouli, Humidah Alanazi, Abir Ben Bacha

**Affiliations:** 1 Laboratoire de Biochimie et de Génie Enzymatique des Lipases, ENIS Route de Soukra, Université de Sfax-Tunisia, Sfax 3038, Tunisia; aida.karray@enis.tn; 2 Biochemistry Department, Science College, King Saud University, P.O. Box 22452, Riyadh 11495, Saudi Arabia; moalonazi@ksu.edu.sa (M.A.); hialanazi@ksu.edu.sa (H.A.); 3 Institut de Pharmacologie de Sherbrooke, Université de Sherbrooke, Sherbrooke, QC J1H 5N4, Canada; jallouliraida@yahoo.fr; 4 Laboratory of Plant Biotechnology Applied to Crop Improvement, Faculty of Science of Sfax, University of Sfax, Sfax 3038, Tunisia

**Keywords:** α-amylase inhibitor, insecticide effects, kinetic parameters, plant defense

## Abstract

The main objective of the current study was the extraction, purification, and enzymatic characterization of a potent proteinaceous amylase inhibitor from *Moringa oleifera*. The antimicrobial potential and insecticide effects against *C. maculates* insect larvae were also studied. The α-amylase inhibitor was extracted in methanol (with an inhibitory activity of 65.6% ± 4.93). Afterwards, the inhibitor αAI.Mol was purified after a heat treatment at 70 °C for 15 min followed by one chromatographic step of Sephadex G-50. An apparent molecular weight of 25 kDa was analyzed, and the N-terminal sequence showed the highest identity level (89%) with the monomeric α-amylase inhibitor from *Triticum dicoccoides*. αAI.Mol was found to tolerate pH values ranging from 5.0 to 11.0 and showed maximal activity at pH 9.0. Thermal stability was remarkably important, since the inhibitory activity was maintained at 55% after 1 h of incubation at 70 °C and at 53% after an incubation of 45 min at 80 °C. The potency of the current purified inhibitor against amylases from different origins indicates that αAI.Mol seems to possess the highest affinity toward human salivary α-amylase (90% inhibitory activity), followed by the α-amylase of insects *Callosobruchus maculatus* and *Tribolium confusum* (71% and 61%, respectively). The kinetic parameters were also calculated, and the K_max_ and V_max_ of the digestive amylase were estimated at 185 (mmol/min/mg) and 0.13 mM, respectively. The inhibitor possesses a strong bactericidal effect against Gram+ and Gram- strains, and the MIC values were >1 against *B. cereus* but >6 against *E. coli.* Interestingly, the rates of survival and pupation of *C. maculates* insect larvae were remarkably affected by the purified αAI.Mol from *Moringa oleifera*.

## 1. Introduction

The regulation of enzymatic activity can be provided by compounds called effectors: activators or inhibitors that act directly or indirectly on the enzyme’s active site. Inhibitors are generally molecules of a similar structure to the substrate, but inhibitors do not react, or they react much more slowly than the substrate [1]. Various natural inhibitor sources, such as plants, microalgae, and macroalgae, are available. In fact, plants are known for their production of proteins and/or secondary compounds (e.g., phenolic compounds) to prevent insect feeding or several pathogen invasions. Indeed, proteases and amylase inhibitors (Amy.Inh) are known to function as plant defense molecules against pests and pathogens. Such compounds are capable of inhibiting the activity of amylases in mammals and insects, but lack activity against amylases of plant and microbial origin. Its activity makes it an interesting insecticide, which can oppose the development of beetle larvae responsible for considerable losses in the world’s seed reserves [2,3]. The enzyme inhibitors act on key insect gut digestive hydrolases, the α-amylases and proteinases. Several kinds of α-amylase and proteinase inhibitors, present in seeds and vegetative organs, act to regulate the numbers of phytophagous insects [4,5,6].

Proteinaceous α-amylase inhibitors are widely present in microorganisms, plants, and animals [7]. Interestingly, proteinaceous inhibitors from plants are largely present in cereals, such as wheat (*Triticum aestivum*) [8,9] and barley (*Hordeum vulgareum*) [10], but also in leguminosae, such as pigeonpea (*Cajanus cajan*) [11] and cowpea (*Vigna unguiculata*) [12]. Structurally, Amy Inhi has been found to be monomeric with molecular masses of 5 kDa [13], 9 kDa [12], and 13 kDa [14], dimeric (homo and heterodimeric) with masses of 26 kDa [14,15], and tetrameric masses of about 50 kDa [16]. α-Amylase inhibitors from plants reveal distinct specificities against digestives amylases from different sources. The specificities of inhibition are crucial to discovering new and useful inhibitors for generating insect-resistant transgenic plants. Research on plant proteinaceous inhibitors of amylases from storage pests has greatly increased recently. Several studies have indicated the potential role of these inhibitors as components of the plant resistance mechanism to pests [17]. A potent amylase inhibitor from *A. aspera* was partially purified. Feeding analysis confirmed the effectiveness of the inhibitor of *A. aspera* against the storage pest *C. maculates* [17]. The isolated inhibitor inhibited the majority of amylase isoforms of *C. maculatus*, *Tribolium confusum*, and *Helicoverpa armigera* in electrophoretic analysis and solution assays [17].

In the last few decades, several studies have been conducted on the application of moringa plants. *Moringa oleifera* is a fast-growing, drought-resistant tree of the family Moringaceae, native to the Indian subcontinent. Common names include the moringa, drumstick tree, horseradish tree, and ben oil tree or benzolive tree. In fact, it has been reported that the root part of the Moringa plant exhibits anti-inflammatory and anti-nociceptive effects. Moringa has also been reported to show hepatoprotective and antibiotic activity. Additionally, the ethanol extract of the Moringa leaf was also reported to have chemopreventive, antioxidant, antispasmodic, cholesterol-lowering antidiuretic, and antifungal activity [18,19,20,21,22]. The potential role of the Moringa leaf as a proteinaceous α-amylase inhibitor has a chance of being developed, due to the content of amino acids and some secondary metabolite compounds contained within them. These inhibitor genes could be considered useful tools for the improvement of crop protection, and their effectiveness when expressed in heterologous plants.

This study aimed to evaluate the activity of amylase inhibitor extracted from moringa leaves against some important digestive amylases, in addition to their antimicrobial and insecticide potential.

## 2. Results

### 2.1. Extraction of the α-Amylase Inhibitor from Moringa oleifera (αAI.Mol) Solvent Optimization

As shown in Table 1, extractions prepared from the leaf processed 45% of the α-Amylase inhibitory activity. The root extracts processed 33%, followed by the fruit, flower, and seed aqueous extract, with 23%, 18%, and 14%, respectively.

**Figure 1:**
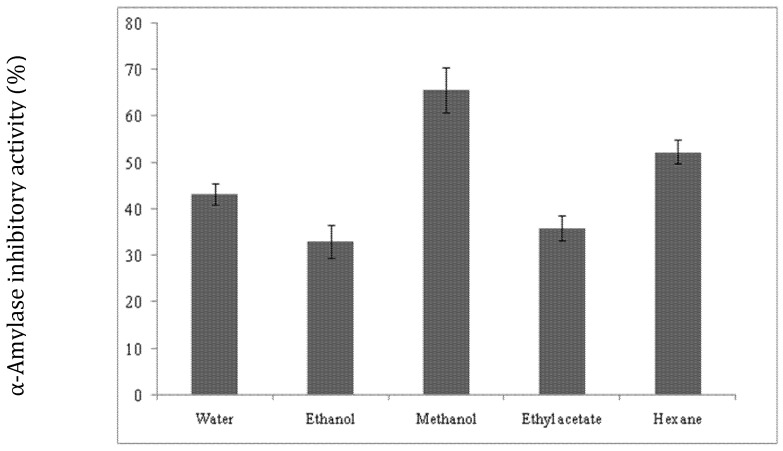
α-Amylase inhibitor activity extracted from *Moringa oleifera* using different solvents.

The resulting soluble crude extracts prepared in methanol showed the maximum α-amylase inhibitor activity (65.6% ± 4.93), which was followed by that prepared in hexane (52.3% ± 2.5) (Figure 1). Much less amylase inhibitor activity was found with extracts prepared in distilled water, ethylene acetate, and ethanol, with inhibition rates of 43.3% ± 2.3, 36% ± 2.6, and 33% ± 2.6, respectively.

### 2.2. Purification of α-Amylase Inhibitor from Moringa oleifera (αAI.Mol)

The purification flow sheet is summarized in Table 2.

**Figure 2:**
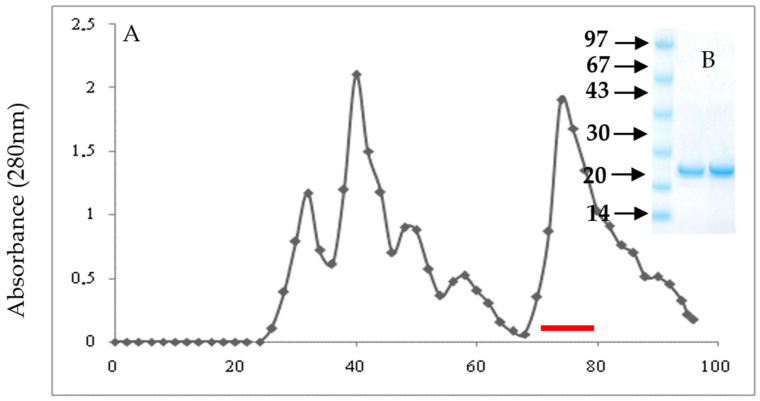
Purification of αAI.Mol: (**A**) gel filtration chromatography on Sephadex G-50. Column of G-50 (2.5 × 100 cm) equilibrated with 0.1 Μ Tris-HCL buffer, pH 8, containing 0.2 Μ NaCl. The column was eluted with the same buffer at a flow rate of 30 mL/h, and 4.55 mL fractions were collected. Red line: active fractions. (**B**) SDS-PAGE analysis. Twenty micrograms of the active fractions containing the amylase inhibitor activity.

Figure 2A represents the elution profile of gel filtration through a Sephadex G-50 column (2.5 × 100 cm) equilibrated with a 0.1 Μ Tris-HCL buffer, pH 8, containing 0.2 Μ NaCl. The elution was performed with the same buffer at a flow rate of 30 mL/h, and 4.55 mL fractions were collected. The active fractions containing αAI.Mol activity appeared asa distinct peak at 1.8 void volumes (Figure 2A). The purification fold of αAI.Mol reached 141.6, with a recovery of 35%. The specific activity was 11,890 IU/mg. The SDS-PAGE of the pure analyzed molecule (Figure 2B) shows that αAI.Mol possesses an apparent molecular mass of about 25 kDa. The amino acid sequence of the protein external NH2 presented in the first 50 residues was determined: SGPWSWCDPA AVKYVSALTG CRAMVKLECV GSEVPEAAIR DCCEQIADLN.

### 2.3. Biochemical Characterization of Pure αAI.Mol

#### 2.3.1. Effect of pH and Temperature on the αAI.Mol Activity and Stability

**Figure 3:**
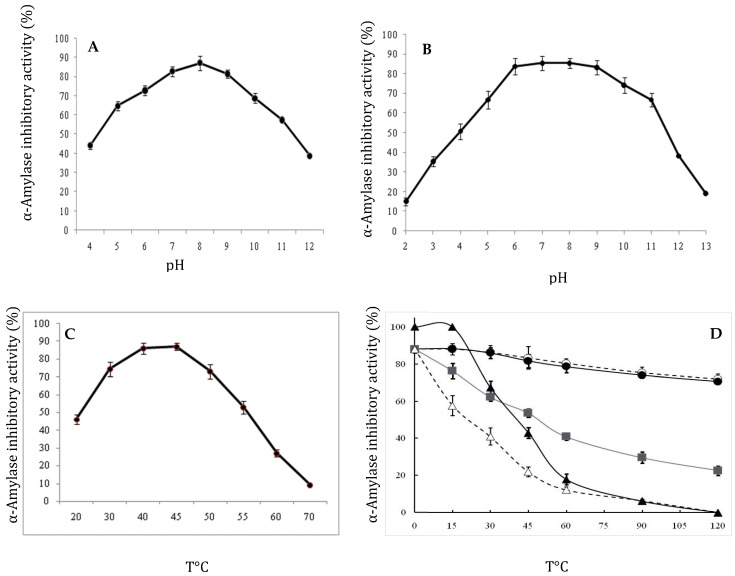
(**A**,**B**) Effect of pH and temperature on αAI.Mol activity: Effect of pH on PDInhibitor activity (**A**) and stability (**B**). Inhibitor activity was assayed at various pH levels, and inhibitor stability was tested after an incubation of the αAI.Mol at different pH levels for 12 h. (**C**,**D**) Effect of temperature on αAI.Mol activity (**C**) and stability (**D**). Inhibitor activity was assayed at various temperatures (50 °C–90 °C). For stability studies, the amylase inhibitor was incubated at different temperatures and drawn at various time intervals and assayed for residual inhibitor activity at optimal conditions of pH and temperature. Data shown are mean ± SD (*n* = 3).

The effects of pH on αAI.Mol activity and stability were performed as described in the Methods section. Figure 3 shows that the maximum α-amylase inhibitor activity was observed at pH 8 (87% ± 3.6% inhibition). Nearly the same amounts were observed at pH 7 and 9, at 81% and 83%, respectively (Figure 3A). Under acidic conditions, we noticed a decrease in inhibitory activity at pH 4.0 to 44%. Moreover, we noticed that the stability of αAI.Mol was maintained at pH values ranging from 6 to 9 (Figure 3B). A stability decrease of about 50% was observed at acidic pH values (pH 4). Moreover, the inhibitor maintained more than 77% of its activity at pH 11 for 12 h (Figure 3B).

Concerning the temperature studies, the effects of various temperatures on pure amylase inhibitor activity and stability were studied using the standard assay. Results from Figure 3C confirm that αAI.Mol was active at temperatures ranging from 30 to 60 °C, but at various rates. In fact, the maximal amylase inhibition activity of 87% ± 2 was observed at 50 °C. Interestingly, αAI.Mol maintained 50% of its inhibitory activity at 60 °C. Moreover, we can conclude from Figure 3D that thermal stability was not affected, since αAI.Mol maintains 78% of its activity at 60 °C and 50% of it at 70 °C for 1 h.

#### 2.3.2. Effect of Various Divalent Ions on α-Amylase Inhibitor Activity

**Figure 4:**
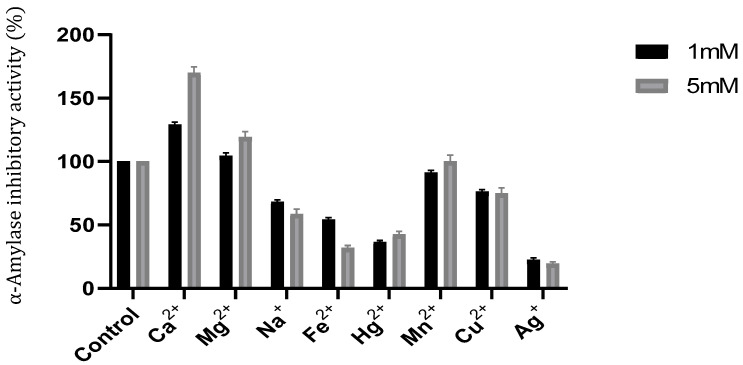
Effects of metal ions at concentrations of 1 and 5 mM on αAI.Mol activity. The α-amylase inhibitor assay was performed at 45 °C and pH 8. The control represents 100% of the α-amylase inhibitor activity under the same condition in the absence of any metal. Data shown are mean ± SD (*n* = 3).

Several divalent metal ions support a key role in maintaining the structural integrity of the tertiary structure of cysteine amylase inhibitors. The inhibitory activity of αAI.Mol was measured in the presence of 1 and 5 mM metal ions and compared with a control (100%) measured at the same conditions but in the absence of metal ions. Figure 4 shows remarkably that Ca^2+^ and Mg^2+^ ions at 5 mM improves αAI.M coinhibitory activity by up to 169.98% and 119%, respectively. Conversely, divalent ions such as Fe^2+^, Na^+^ and Hg^2+^ at 5 mM were noted to reduce inhibitor activity by up to 31%, 58%, and 42%, respectively. Mn^2+^ maintains the same initial inhibitor activity. Na^+^ reduces the inhibitor activity by 68% and 58% at 5 and 1 mM, respectively.

#### 2.3.3. Activity of α-Amylase Inhibitors from *Moringa oleifera* against Mammalian, Bacterial, and Insect α-Amylases

The α-amylase inhibitor from *Moringa oleifera*, named αAI.Mol, was tested against seven available amylases from different organisms (porcine pancreatic, human salivary, *Bacillus subtilis*, *Aspergillus oryzae*, *Bacillus pacificus*, *Tribolium confusum*, and *Callosobruchus maculates*) (Table 3). αAI.Mol seems to possess the highest affinity toward human salivary α-amylase (90% inhibitory activity), followed by the α-amylase of insects *Callosobruchus maculatus* and *Tribolium confusum* (71 and 61%, respectively). Moreover, the α-amylase inhibitor exhibited much less affinity toward *Aspergillus oryzae* (16%).

### 2.4. Antimicrobial Activity of the Purified αAI.Mol

**Figure 5:**
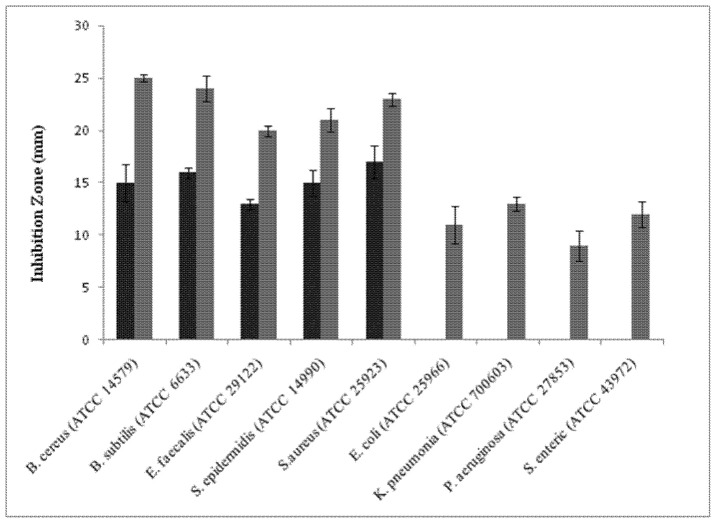
Antibacterial properties of the alpha amylase inhibitor (αAI.Mol) from *Moringa oleifera)*. The antibacterial effect was evaluated against several Gram-positive and Gram-negative bacteria presented by zone of inhibition (mm) using pure amylase inhibitor (gray bars) and crude extract (black bars).

An antibacterial assay of the purified amylase inhibitor from *Moringa oleifera* was carried out against bacteria (Gram+ and Gram-bacteria). The bactericidal effect was analyzed by measuring the inhibition zone diameter resulting from the inoculation of bacteria with crude and purified amylase inhibitors, at the same conditions (Figure 5). MIC values were also recorded (Table 4). Figure 5 shows that αAI.Mol possesses a strong bactericidal effect against bacilli strains *B. cereus* (ATCC 14579) and *B. subtilis* (ATCC 6633), but a moderate effect against *E. faecalis* (ATCC 29122) and *S. epidermidis* (ATCC 14990) for either the crude extract or the pure inhibitor. In fact, the diameter inhibition zone ranges from 20 to 25 mm. Gram-negative strains were more resistant to αAI.Mol than the pure inhibitor with a diameter inhibition zone of 15 mm. The MIC values were estimated to be >1 against *B. cereus* (ATCC 14579) and *B. subtilis* (ATCC 6633), >2 against *E. faecalis* (ATCC 29122), and *S. aureus* (ATCC 25923), and >3 for *S. epidermidis* (ATCC 14990). For Gram-negative bacteria, the MIC values were >5 against *K. pneumonia* (ATCC 700603), >6 for *E. coli* (ATCC 25966), and >7 against *P. aeruginosa* (ATCC 27853) and *S. enteric* (ATCC 43972) (Table 4).

### 2.5. Insecticide Effects of the Purified αAI.Mol against C. maculates Larvae Insect

**Figure 6:**
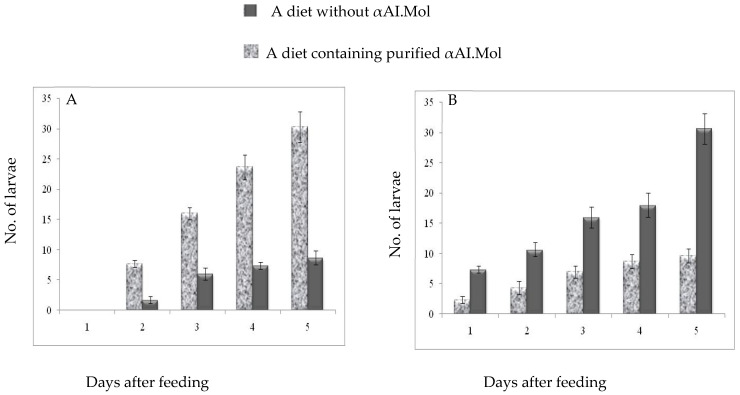
Bioassays of purified amylase inhibitor (αAI.Mol) from *Moringa oleifera* against *C. maculates* insect larvae: pupation and mortality. (**A**) Rate of pupation of *C. maculates* insect larvae. The bioassay was studied by feeding *C. maculatus* larvae a diet containing purified αAI.Mol and compared to a control of *C. maculatus* larvae on a diet without the inhibitor αAI.Mol (gray bars). Pupation was recorded every 24 h and continued for up to 5 days. The experiment was repeated in triplicate, and standard deviations were recorded. (**B**) Rate of mortality of *C. maculates* insect larvae. Mortality of larvae fed the αAI.Mol-containing diet as compared with the control of *C. maculatus* larvae on a diet without the inhibitor αAI.Mol (gray bars) diet was studied for up to 5 days.

The effects of the feeding experiment on pupation and mortality rates were analyzed for 5 days. The results are summarized in Figure 6A,B, respectively. No pupation was observed on the first day of feeding, while the mortality rate increased relative to the control. Indeed, this observation is due to the toxic effect of the inhibitor. On the second day of feeding, it was again observed that the pupation rate decreased by up to 4 times (Figure 6A), and the mortality rate increased by up to 3 times (Figure 6B) for larvae fed on the test diet compared with those fed on the control diet (presented in gray). This model was sustained over the five-day period. Consequently, the rates of survival and pupation were remarkably affected by the purified αAI.Mol from *Moringa oleifera* (Figure 6).

### 2.6. Kinetic Parameters of the Purified αAI.Mol: V_max_ and K_max_

**Figure 7:**
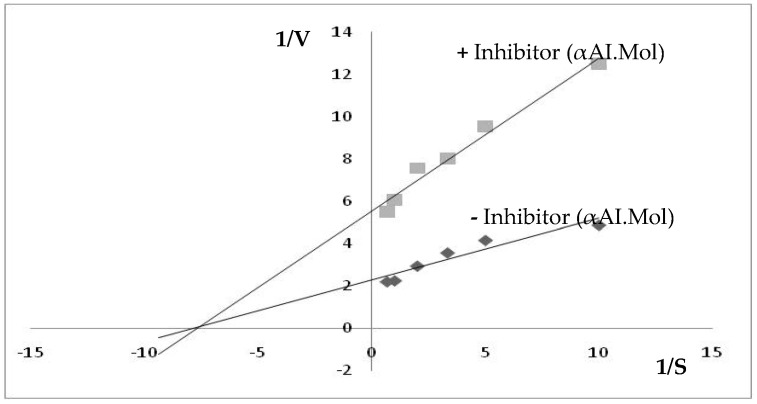
Lineweaver Burk curves: the hydrolysis rates (V) of pure starch (S) at various concentrations [0–1.5 mM] at 45 °C, pH 8, for 15 min using α-amylase incubated with 10 µg of inhibitor (+αAI.Mol: green square) or without the inhibitor (−αAI.Mol: black square).

Lineweaver Burk curves were plotted and confirmed the type of inhibition: noncompetitive (Figure 7). In fact, K_max_ and V_max_ were calculated by the Lineweaver Burk plot. From these fits, the K_max_ and V_max_ of the digestive amylase were estimated at 185 (mmol/min/mg) and 0.13 mM, respectively, when 10 µmol of αAI.Mol was used. The V_max_ of the digestive amylase was 0.33 mM when tested without the inhibitor. The affinity of the enzyme was not clearly affected, indicating noncompetitive inhibition. The curves extrapolation with the abscissa axis permits the deduction of a K_i_ value of about 23 µM.

## 3. Discussion

Pathogens (fungi, bacteria, and viruses) and insect pests are responsible for severe yield losses. In fact, worldwide, losses in agricultural production due to pest attacks are around 37% [23], contrary to pathogens, where the insects feed directly on the plant tissues. Consequently, plants have developed resistance against insect predation. The current resistance is due to a set of defense mechanisms obtained by plants during evolution [24]. These defense compounds may be antibiotics or alkaloids, or could be chitinases, b-1,3-glucanases, and enzyme inhibitors [25,26,27,28]. The latter act on key insect gut digestive hydrolases: the α-amylases and proteases. Numerous kinds of such hydrolase inhibitors, naturally present in seeds and vegetative organs, proceed to control numbers of phytophagous insects [4,5,23]. The current study is focused on the biochemical characteristics as well as the biological effects of a α-amylase purified from *Moringa oleifera*. Amylase inhibitors are widely known for their monomeric form, structurally possessing molecular masses of 5 [13], 9 [12], and 13 kDa [14]. The molecular masses are of about 26 [14] and 50 kDa [16] for dimeric (homodimeric or heterodimeric) and tetrameric 3D structures, respectively. Proteinaceous α-amylase inhibitors are found in different living microorganisms, plants, and animals [25,29] and are composed of a large and diverse family of α-amylase inhibitors. In fact, they are conveniently classified by their tertiary structure into six classes: lectin-like, knottin-like, cereal-type, Kunitz-like, c-purothionin-like, and thaumatin-like. [30]. α-Amylase inhibitors act as protective proteins, and the continuing discovery of new sources and classes of α-amylase inhibitors have aroused great interest in biotechnology and medicine. Herein, we detail the purification steps and biochemical characterization of a new proteinaceous α-amylase inhibitor extracted from the plant *Moringa oleifera*. The biological properties of the inhibitor and its potential against bacteria, fungi, and insects were also studied. We first performed an aqueous extraction from different parts of the *Moringa oleifera* plant. We noticed that extractions prepared from the leaf processed the best rate of α-amylase inhibitor activity (45%), followed by the root extract (33%) and subsequently the fruit, flower, and seed aqueous extracts, with 23%, 18%, and 14%, respectively. From 100 g of Moringa leaves, we tested the suitable solvent permitting a maximal extraction and an improvement in the complete extraction of biomolecules from the plant source. Using a range of extraction solvents and the α-amylase inhibitor from *Moringa oleifera*, we found that extracts in methanol showed maximum α-amylase inhibitor activity (65.6% ± 4.93), which was followed by that prepared in hexane (52.3% ± 2.5). In previous studies, decorticated and defatted seeds of *Achyranthes aspera* have been extracted in distilled water (1:6 w/v) at room temperature for 24 h, and the supernatant was used as a crude inhibitor extract (wileyonlinelibrary.com) [31]. After heat treatment at 70 °C for 15 min, the resulting supernatant containing 47 mg of active α-amylase inhibitor was loaded on a G-50 filtration chromatography device. The obtained pick containing the inhibition activity was analyzed by SDS-PAGE electrophoresis, which clearly indicated that the apparent molecular weight of the α-amylase inhibitor (αAI.Mol) was estimated to be around 25 kDa, suggesting a dimeric structure inhibitor family, characterized by a molecular mass of 26 kDa [14]. The N-terminal sequence of αAI.Mol presented in the first 50 residues was determined: SGPWSWCDPA AVKYVSALTG CRAMVKLECV GSEVPEAAIR DCCEQIADLN. The highest identity level (89%) was observed with a monomeric α-amylase inhibitor in *Triticum dicoccoides* and *Triticum aestivum*, with accession numbers ACQ83718.1 and QLH12252.1, respectively. An identity of 87% was observed with the dimeric α-amylase inhibitor from the plant *Heterantheliumpiliferum* (ACP40911.1). Moreover, αAI.Mol was found to be stable in a large pH range, as it maintains 55% of the inhibition rates at pH values ranging from 5 to 11. The inhibitor was found to be active at pH values ranging from 5.0 to 11.0, with maximal activity at pH 9.0 (87% inhibition). Interestingly, αAI.Mol was found to express its full activity at 50 °C and maintained 90% of its stability at over 55 °C. In fact, thermal stability was remarkably important, since the inhibitor activity was maintained at 55% after 1 h of incubation at 70 °C, and at 53% after 45 min incubation at 80 °C. pH and thermal stability are considered interesting and promising features of α-amylase inhibitors, as they can act as components of pest resistance mechanisms. In fact, tolerance to extreme temperatures and pH values indicate the efficiency of inhibitors against a range of phytophagous insects at special gut conditions. In previous work, α-amylase inhibitors from Beni Suef-1 and Beni Suef-5 varieties [32] were found to be stable at temperatures below 80 °C and in a wide pH range (2–12). Maximum activity was obtained at 40–50 °C. Ca^2+^ (10 mM) was found to be the best activator of the inhibition activity of purified αAI.Mol, followed by Mg^2+^ (10 mM), with an increase in inhibition activity of 69% and 20%, respectively. Ca^2+^ ions probably affected the site of the inhibitor’s binding to the α-amylase enzyme. Afterwards, we studied the potency of the current purified inhibitor against the amylase from different origins. αAI.Mol seems to possess the highest affinity toward human salivary α-amylase (90% inhibitor activity), followed by the α-amylase of insects *Callosobruchus maculatus* and *Tribolium confusum* (71 and 61%, respectively). Moreover, α-amylase inhibitors exhibited much less affinity toward *Aspergillus oryzae* (16%). In a previous work on insect amylases, it has been reported that Beni Suef-1 and Beni Suef-5 α-amylase inhibitors have higher affinity (with lower Ki values) towards the α-amylase of *T. castaneum* than that of *C. maculates* [32]. The specificity of the inhibitor against such α-amylases is probably due to the amino acid differences between insect and mammalian enzymes at the interface, leading to a reduced hydrogen-bonding capability, in the absence of any obvious steric impediment to the formation of the enzyme/inhibitor complex [33].

To better characterize the enzymatic inhibition, kinetics analyses were conducted and revealed that the αAI.Mol α-amylase inhibitor is a noncompetitive α-amylase inhibitor, with a K_max_ and V_max_ of the digestive amylase of about 185 (mmol/min/mg) and 0.13 mM, respectively. To our knowledge, the current work is the first study dealing with the kinetic parameters of noncompetitive α-amylase inhibitors. The biological activities of proteinaceous α-amylase inhibitors are of great interest, especially their potential against pathogenic microorganisms or organisms that are resistant to certain antibiotics, and their effectiveness in biological mechanisms for insect control [34,35,36]. The current results indicate that αAI.Mol possesses a strong bactericidal effect against bacilli strains *B. cereus*(ATCC 14579) and *B. subtilis* (ATCC 6633), but a moderate effect against *E. faecalis* (ATCC 29122) and *S. epidermidis* (ATCC 14990) for both crude extracts and pure inhibitors. Gram-negative strains were more resistant to αAI.Mol compared to the pure inhibitor. In fact, MIC values were about >5 against *K. pneumonia* (Gram-negative) but >1 against *B. cereus* and *B. subtilis* (Gram-positive) when the pure inhibitor was used. The antibacterial effect of the amylase inhibitor is attributed to its role as a regulator of endogenous enzymes. Several phenolic compounds are endowed with α-amylase inhibitory activity. The Cu^2+^, Fe^2+^, and Hg^2+^ ions are therefore competitive inhibitors (analogs structural to activators) [37]. Moreover, some α-amylase organic inhibitors have been studied, such as maltose, which is the hydrolysis product of starch from an amylase that leads to a feedback inhibition enzyme [37].

It is well established that inhibitors from plants are thought to make them protective and even lethal to insects [38]. In fact, proteinaceous α-amylase inhibitors are under further in vitro and in vivo antimetabolic evaluation for their effects on *T. castaneum* [39,40].

The current study indicates that the mortality rate of *C. maculates* insect larvae was increased compared to the control *C. maculates* larvae fed without an amylase inhibitor. The same behavior was observed in the bioassays: a strong decrease in the pupation rate but an increase in the mortality rate of insect larvae fed on a test diet. The same model was maintained over the 5-day period of the test. Consequently, the rates of survival and pupation were remarkably affected by the purified αAI.Mol from *Moringa oleifera.* Analysis ofα-amylase inhibitors from wheat, barley, and millet showed high potency against coleopteran insects [41]. The decrease in the pupation rate may be due to the inhibition of amylases that are essential for providing the energy source required for active pupation. A previous study indicates that the amylase inhibitor could be a good candidate for conferring resistance against insects that mainly depend on amylolytic enzymes for their survival. Feeding analysis confirmed the effectiveness of the α-amylase inhibitor of *A. aspera* against the storage pest *C. maculatus*. These observations indicate that the incorporation of a purified α-amylase inhibitor in a diet affects the rate of survival and the rate of pupation of larvae as compared with the control [33].

The current study could be significant for understanding the molecular mechanisms involved in the ecology of plant–herbivore interactions. Thus, the inhibitor genes could be considered useful tools for the improvement of crop protection and their effectiveness when expressed in heterologous plants.

## 4. Materials and Methods

### 4.1. Extraction of α-Amylase Inhibitor

The barks, fruits, leaves, roots, and seeds of *Moringa oleifera* plants were randomly collected in March 2020 from nearby areas around King Saud University in Riyadh city (Kingdom of Saudi Arabia). The plant was identified and confirmed by Dr. Mona S. Alwahibi (Botany and Microbiology Department, Science College—King Saud University). Plant tissues were first washed in distilled water, cut into small pieces for easy air-drying at ambient temperature, and ground. Afterwards, 20 g of each powdered tissue was homogenized in 200 mL of different solvents including water, ethanol, ethyl acetate, hexane, or methanol. Following 24 h incubation in a rotary shaker at 200 rpm and at room temperature, homogenates were filtered using a Buchner funnel and centrifuged (12,000 rpm, 15 min, 4 °C) and concentrated using a rotary evaporator. Thereafter, resulting materials were properly dried on a laboratory bench before being stored at 4 °C for further use.

### 4.2. α-Amylase Inhibitor Activity Assay

α-Amylase inhibitor activity was investigated using starch as a substrate according to Bernfeld’s method [42]. A starch solution was prepared in a 0.1 M Tris-HCL buffer, pH 8, containing 0.1 mM CaCl_2_ and 20 mM NaCl. One α-amylase unit (UI) was defined as the amount of this enzyme that increased absorbance by 0.1 OD at 530 nm during 25 min of the assay. For comparative purposes, amylase inhibitor activity was also expressed as an inhibition percentage, which was calculated by comparison with a control experiment. All experiments were carried out in triplicate.

### 4.3. Enzymes

Human salivary α-amylase (HSA), porcine pancreatic α-amylase (PPA), *Bacillus subtilis (BSA)*, and *Aspergillus oryzae (AOA)* were purchased from Sigma Co., St. Louis. *Bacillus pacificus*α-amylase (BPA) was purified as recently reported by Alonazi et al. (2020), and the two larval insect α-amylases from *Tribolium confusum* (AOA) and *Callosobruchus maculatus* (CMA) were obtained, as previously described by Hivrale et al. (2011).

### 4.4. Purification of Amylase Inhibitor

The crude extract (70 mL, 188,900 IU) was first subjected to fractionation using ammonium sulfate (40–85%) and centrifuged (12,000 rpm, 45 min, 4 °C), and the obtained precipitate was resuspended in 15 mL of 0.1 M Tris-HCL buffer (pH 8). Thereafter, the resulting supernatant (20 mL, 151,050 IU) was further fractionated by 40–85% saturation with ethanol. Precipitated proteins containing about 67% of the starting amount of the amylase inhibitor were dissolved in a minimum of 0.1 M Tris-HCL buffer (pH 8), and the collected supernatant (15 mL, 126,790 IU) was incubated at 70 °C for 15 min and then rapidly cooled and centrifuged (12,000 rpm, 30 min, 4 °C).

The resulting crude inhibitor preparation (15 mL, 115,855 IU) was loaded onto a Sephadex G50 column (2.5 × 100 cm) pre-equilibrated with a 0.1 M Tris-HCL buffer (pH 8) containing 0.15 M NaCl. The same buffer was used for protein elution, and 4.5 mL fractions were collected at a flow rate of 0.5 mL/min. The protein elution profile was followed spectrophotometrically at 280 nm. Fractions containing amylase inhibitor activity were analyzed electrophoretically by SDS-PAGE (15%), and pure active fractions were then gathered and stored at 4 °C for further analysis.

### 4.5. Protein Analysis

Protein content was determined according to the method of Bradford [43]. The purity and the molecular weight estimation of the studied amylase inhibitor were checked with 15% SDS-PAGE following the method of Laemm Li [44] (1970). NH_2_-terminal sequence was determined by automated Edman’s degradation using an Applied Biosystems Protein Sequencer Procise 492 equipped with a 140 C HPLC system [45].

### 4.6. Effect of pH and Temperature on the Amylase Inhibitor Activity and Stability

Thermal stability of the purified amylase inhibitor was investigated by incubating the inhibitor in a water bath at different temperatures, ranging from 50 to 90 °C and at different time intervals (15, 30, 45, 60, 90, and 120 min). After rapid cooling at 4 °C for 10 min followed by centrifugation (10,000 rpm, 10 min, 4 °C), the residual α-amylase inhibitor activity of the treated inhibitors was determined with time, as described earlier. Furthermore, the optimum temperature of the amylase inhibitor activity was determined by carrying out the enzyme assay at different temperatures (20–80 °C) at pH 8.

On the other hand, the effect of pH on the inhibitor activity of the purified α-amylase inhibitor was checked at different pH ranges (from 2–12) using the following buffers: a glycine-hydrochloric buffer at pH 2, a citrate-phosphate buffer at pH 3–5, a phosphate buffer at pH 6–8, a glycine-NaOH buffer at pH 9–11, and potassium chloride-NaOH at pH 12–13. The purified α-amylase inhibitor was also incubated in a buffer solution (pH 2–13) for 2 h at ambient temperature, and the residual α-amylase inhibitor activity was determined as described earlier. All experiments were performed in triplicate.

### 4.7. The Metal Ion Effect on the Amylase Inhibitor Activity

The effect of metal ions on the amylase inhibitor activity was investigated by the addition of various divalent ions (including Ca^2+^, Mg^2+^, Na^+^, Fe^2+^, Hg^2+^, Mn^2+^, Cu^2+^, and Ag^+^) to the reaction medium at a final concentration of 1 or 5 mM. After incubation for 1 h at 40 °C, the amylase inhibitor activity was measured under optimal conditions as described above.

### 4.8. Inhibitory Activity of the Purified Protein in Amylases from Different Organisms

The activities of seven available amylases from different organisms (porcine pancreatic, human salivary, *Bacillus* *subtilis*, *Aspergillus* *oryzae*, *Bacillus* *pacificus*, *Tribolium confusum*, and *Callosobruchusmaculates* were determined according to previously reported protocols. The enzyme inhibition using αAI.Mol (0.25 mg/mL) was determined after preincubation for 15 min. Afterwards, the remaining enzyme activity was measured. αAI.Mol activity on the respective enzyme is expressed as the percentage of inhibition.

### 4.9. Antimicrobial Activity of αAI.Mol

The antibacterial effect of the purified amylase inhibitor was evaluated against the following strains: Gram-positive *Bacillus* cereus (ATCC 14579), *Bacillus* subtilis (ATCC 6633), *Staphylococcus* aureus (ATCC 25923), *Staphylococcus* epidermidis (ATCC 14990), and *Enterococcus* faecalis (ATCC 29122) and Gram-negative *Escherichia* coli (ATCC 25966), *Salmonella* enteric (ATCC 43972), *Pseudomonas* aeruginosa (ATCC 27853), and *Klebsiella* pneumonia (ATCC 700603) using the agar diffusion method. The bacterial strains were cultured for 24 h in a nutriment broth. Two hundred microliters of each bacterial suspension (106 CFU) was spread on Luria broth agar, and pores were then loaded with 10 µL of pure αAI.Mol (1 mg/mL) filtered through a 0.22 mm Millipore filter beforehand. The plates were incubated overnight at 37 °C. A 0.1 Μ Tris-HCL buffer, pH 8, was used as the negative control. The inhibition zones were measured (in millimeters) on the surface of the top agar in triplicate, and the averages of three separate assays were reported. Afterwards, the micro-well dilution method was used to determine the lowest compound concentration (MIC) that completely blocks the growth of tested microorganisms. Dilution series of the tested enzyme (0.5–20 µg/mL) were set in a 96-well plate as described by Andrews [46]. In each well, a mixture consisting of 50 µL of the diluted compound, 10 µL of an inoculum, and 40 µL of a growth medium was incubated for 24 h at 37 °C. Afterwards, 40 µL of MTT (0.5 mg/mL) was added to each well, and the plate was again incubated for 30 min at the same temperature. The well showing no change to a violet-colored formazan compound indicated that the bacteria were biologically inactive and correspond to the MIC.

### 4.10. Rearing of Insects and Dietary Studies

*C. maculatus* and *T. confusum* specimens were collected from rice and chickpeas, respectively, and reared under a 12/12 h light/dark photoperiod at a relative humidity of 70% and a constant temperature of 28 °C. The insects were fed on rice or chickpeas until further use. However, the bioassays were performed by feeding *C. maculatus* larvae on a test diet containing a pure amylase inhibitor (10 g/kg) incorporated into defatted chickpea powder or on a control diet consisting of only defatted chickpea powder. Twenty early second-instar larvae were reared on this diet, and their rates of pupation and mortality were recorded at 24 h intervals. The assay was started at time zero and continued for up to 5 days. The larvae surviving after the first day continued to be fed on the same diet on the second day, and so on. The pupation rate was calculated from the total number of pupae and the larval growth rates were compared. The experiment was repeated at least three times in triplicate [46].

### 4.11. Kinetic Parameters

Amylase activity was evaluated at various substrate concentrations ranging from 0 to 1.5 mM of starch under optimal conditions (pH 8, 45 °C) without an inhibitor and subsequently with 10 µmol of αAI.Mol inhibitor. Measurements were recorded in duplicate, and the respective kinetic parameters, including Vmax (the maximal velocity) and (affinity) Km_app_, were calculated from Lineweaver–Burk plots (Lineweaver and Burk, 1934). The inhibition constant (K_i_) value was determined from the curve extrapolation with the abscissa axis, where *Vmax* is the maximal velocity, and [E] is the active enzyme concentration.

### 4.12. Statistical Analysis

The IBM statistical package for the social sciences software package version 19.0 (IBM corp., Armonk, NY, USA) was used to perform the statistical analysis. All experiments were carried out at least three times and results were expressed as mean ± standard deviation (SD). Statistical significance was reviewed using Student’s t-tests to verify the significance of the observed differences in amylase activities as well as rates of mortality and pupation of *C. maculatus* larvae after treatment with αAI.Mol. The results were considered statistically significant for P values of less than or equal to 0.05.

## Figures and Tables

**Table 1:** Aqueous extract from different parts of the *Moringa oleifera* plant.

Plant Parts	α-Amylase Inhibitory Activity (%)
Leafe	45.33 ± 3.51
Seed	14 ± 2.64
fruit	23 ± 2
flower	18.33 ± 2.08
root	33 ± 3

**Table 2:** Flow sheet of *α*-amylase inhibitor purification.

Purification Step	Total Activity (Units)	Protein (mg)	Specific Activity (U/mg)	Activity Recovery (%)	Purification Factor
Crude Extract	188,900	2250	83.95	100	1
Ammonium Sulphate Fractionation (40–85%)	151,050	940	201	80	2.4
Ethanol Fractionation (50–90%)	126,790	230	551	67	6.56
Heat Treatment (70 °C, 15 min)	115,855	47	2465	61	29.4
Sephadex G50	65,395	5.5	11890	35	141.6

**Table 3:** Activity of α-amylase inhibitors from *Moringa oleifera* against mammalian, bacterial, and insect α-amylases.

Amylases	Amylase Inhibitory Activity (%)	SD
Porcine pancreatic	64	3.60
Human salivary	90.33	3.05
*Bacillus subtilis*	53.33	4.16
*Aspergillus oryzae*	16.66	2.08
*Bacillus pacificus*	52.66	3.51
*Tribolium confusum*	61.66	4.041
*Callosobruchus maculatus*	71.66	3.51

**Table 4:** Antimicrobial activity of αAI.Mol on bacterial strain MIC values.

*Bacterial Strains*	MIC
*B. cereus* (ATCC 14579)	>2
*B. subtilis* (ATCC 6633)	>2
*E. faecium* (ATCC 19433)	>3
*S. aureus* (ATCC 25923)	>3
*S. epidermidis* (ATCC 14990)	>3
*E. coli* (ATCC 25966)	>6
*P. aeruginosa* (ATCC 27853)	>7
*K. pneumonia* (ATCC 700603)	>5
*S. enteric* (ATCC 43972)	>7

## Data Availability

Not applicable.

## Sample Availability

Samples of the compounds are available from the authors.
